# Effect of perineoplasm perinephric adipose tissues on migration of clear cell renal cell carcinoma cells: a potential role of WNT signaling

**DOI:** 10.18632/oncotarget.10467

**Published:** 2016-07-07

**Authors:** Xiaolin Zi, Achim Lusch, Christopher A. Blair, Zhamshid Okhunov, Noriko N. Yokoyama, Shuman Liu, Molly Baker, Victor Huynh, Jaime Landman

**Affiliations:** ^1^ Department of Urology, University of California, Irvine, Orange CA 92868, USA

**Keywords:** migration, proliferation, fat, renal cell carcinoma, WNT

## Abstract

To investigate the cellular and molecular interactions between clear-cell renal cell carcinoma (ccRCC) and perinephric adipose tissue (PAT), perineoplasm PAT, PAT away from the neoplasm, renal sinus and subcutaneous adipose tissues were collected at the time of renal surgery for renal masses and conditioned medium (CM) was generated from 62 patients. Perineoplasm PAT CMs from 44 out of 62 (about 71%) of patients with ccRCC or benign renal diseases (e.g. oncocytomas, angiomyolipomas, multicystic kidney, interstitial fibrosis, etc.) enhanced the migration of CaKi-2 cells. Perineoplasm PAT CMs from ccRCC significantly increased migration of ACHN and CaKi-2 cells by ~8.2 and ~2.4 folds, respectively, relative to those from benign renal diseases, whereas there is no significant difference in migration between ccRCC and benign renal diseases in CMs collected from culturing PAT away from neoplasm, renal sinus and subcutaneous adipose tissues. High Fuhrman Grade was associated with increased migration of Caki-2 cells by perineoplasm PAT CMs. Perineoplasm PATs from pT3 RCCs overexpressed multiple WNTs and their CMs exhibited higher WNT/ß-catenin activity and increased the migration of Caki-2 cells compared to CMs from benign neoplasms. Addition of secreted WNT inhibitory factor-1 recombinant protein into perineoplasm PAT CMs completely blocked the cell migration. These results indicate that WNT related factors from perineoplasm PAT may promote progression of local ccRCC to locally advanced (pT3) disease by increasing ccRCC cell mobility.

## INTRODUCTION

The prevalence of obesity in the United States has increased significantly [1]. This increase in obesity is thought to partly contribute to the steadily increasing incidence of renal cell carcinoma (RCC) over the past two decades [2]. Using body mass index (BMI) > 30 kg/m^2^ [(weight in kg) / (height in m)^2^] as an indicator for obesity, it was estimated that obesity is associated with more than 30–40% of RCC cases. [3]

Higher BMI is associated with greater mass of adipose tissue, which potentially leads to an increased risk of RCC through chronic tissue hypoxia, increased inflammatory response, altered metabolism and endocrine derangements [4]. However, BMI is a value derived from body height and weight for measurement of body fat, which includes fat, muscle, bone, and other tissues. As such, BMI cannot accurately predict the amount of adipose tissue mass at individual levels. In addition, there are different types of adipose tissues (ATs) (*i.e.* visceral and subcutaneous fat) in the body. The kidney is uniquely surrounded by perinephric adipose tissue (PAT) which lies between the capsule of the kidney and Gerota's fascia [5]. RCC can spread into PAT [6–9] and may interact with PAT to dynamically exchange metabolites, cytokines and growth factors. Secreted factors from PAT may affect proliferation, migration, and invasion of neighboring tumor cells. On the other hand, neighboring cancer cells may reprogram adipocytes into fibroblast-like cells to promote expression of MMP11 and cancer cell survival and invasion [10]. These findings suggest that cross talk between adipose tissues and RCC is a complex two-way interaction. Therefore, in order to understand the biological mechanisms of obesity in progression of RCC, there is a need to investigate the direct biological interaction between perineoplasm PAT and RCC.

Additionally, several case-control and clinical case series studies have investigated the relationship between RCC histologic subtypes and obesity [11–14]. These results showed that obesity was associated with clear cell and chromophobe RCC, but not papillary RCC [14]. Consistent with the above reports, we have observed an association of PAT with RCC subtypes in a clinical study of 250 patients with cT (1a) renal cortical neoplasms [5]. PAT was a superior indicator in comparison to other body fat metrics for predicting clear-cell RCC (ccRCC) histopathology [5]. Furthermore, perirenal fat invasion is an important pathological feature of locally advanced pT3 ccRCC and associated with poor prognosis in ccRCC patients [6, 7].

Despite the above findings, the underlying biological processes for the association between PAT and ccRCC remain largely unknown. The objective of the present study is to investigate whether factors, which are secreted by PAT from different Fuhrman grades and from different tumor stages of ccRCC, affect the biological behaviors (*i.e.* cell proliferation and migration) of ccRCC cells. PATs from total or partial nephrectomy with benign renal diseases were used as controls. Since the wingless type (WNT)/β-catenin signaling pathway has been reported as one of the most important regulators for both adipogenesis and renal tumorigenesis [15–17], we investigated the WNT activity of perineoplasm PAT conditioned media (CM) and its correlation with proliferation and migration of renal cancer cells as the initial effort in a planned series of investigations.

## RESULTS AND DISCUSSION

### Clinical and pathological characteristics of patients with ccRCC or benign renal diseases

The clinical and pathological features of 46 ccRCC patients and 16 benign renal diseases, who underwent neoplasm excision surgery from 2012 to 2015 in the Department of Urology at University of California, Irvine are summarized in Table 1. Benign renal diseases included multicystic kidney (n = 1), interstitial fibrosis (n = 2), and hydronephrosis with cystic dilation (n = 2), oncocytoma (n = 4), angiomyolipoma (n = 3), complex renal cysts (n = 3), and benign cysts (n=1). In the current study, ccRCC patients with different tumor stages (pT1, pT2 and pT3) were diagnosed at a similar age. There is also no statistically significant difference in age among different tumor stages of ccRCC and benign renal diseases (*P*>0.05) (Table 1). There were more female patients with benign renal diseases (Table 1).

**Table 1:** Clinical and pathological characteristics of ccRCC and benign renal diseases

Pathology stages	Clear cell RCC			Benign
	pT1	pT2	pT3	
Sex				
Male, n (%)	12 (50)	6 (75)	10 (71.4)	3 (18.7)
Female, n (%)	12 (50)	2 (25)	4 (29.6)	13 (81.3)
Age (mean ±SD)	62 ± 10	63 ± 12	63 ± 10	58 ± 12
BMI (mean ±SD)	29.9 ± 7.8	28.9 ± 4.6	29.2 ± 4.2	27.4 ± 6.6
Tumor volume (mean ±SD)	27.2 ± 38.5	179.8 ± 122	152.6 ± 242.5	28.6± 41.5
Fuhrman grade, n (%)				
1	6 (25)	0 (0)	1 (7.1)	
2	15 (62.5)	3 (37.5)	6 (42.9)	
3	2 (8.3)	1 (12.5)	6 (42.9)	
4	1 (4.2)	4 (50)	1 (7.1)	
**Total (n)**	**24**	**8**	**14**	**16**

The difference of mean BMIs among patients with different tumor stages (pT1, pT2 and pT3) of ccRCC and benign renal diseases was also not statistically significant (*P*>0.05). Patients with pT2 and pT3 ccRCC have larger tumor volumes than those with pT1 or with benign renal diseases (*P*s<0.05).

### Perineoplasm PAT CMs have higher capacity of promoting migration of RCC cells than CMs from other AT cultures, including PAT away from neoplasms, sinus and skin AT

We have recently shown that age, PAT volumes and peripherally located tumors are significant indicators for ccRCC (an aggressive type of RCC) histopathology [5]. Also PAT was superior to other body fat metrics for predicting ccRCC in a multivariate logistic regression analysis of clinical data from 250 patients undergoing renal surgery [5]. Since PATs and peripheral kidney tumors are closely located, there is possibility that PATs may directly interact with peripheral tumors to affect RCC development and progression. To provide a biological explanation for the association between PAT and ccRCC, CMs were collected from different AT cultures, including perineoplasm PAT, PAT away from neoplasms, sinus and skin ATs from patients with ccRCC or benign renal diseases undergoing extirpative renal surgery. RCC lines Caki-2 and ACHN were chosen for studying the effect of CMs of different AT cultures on cell proliferation and migration by the MTT assay and the Boyden chamber cell migration assay, respectively. After these assays, we calculated the percentages of cell proliferation or migration relative to negative controls by dividing the proliferative or migratory values of AT CM by those of the negative controls that were cultured under serum-free medium conditions.

**Figure 1:**
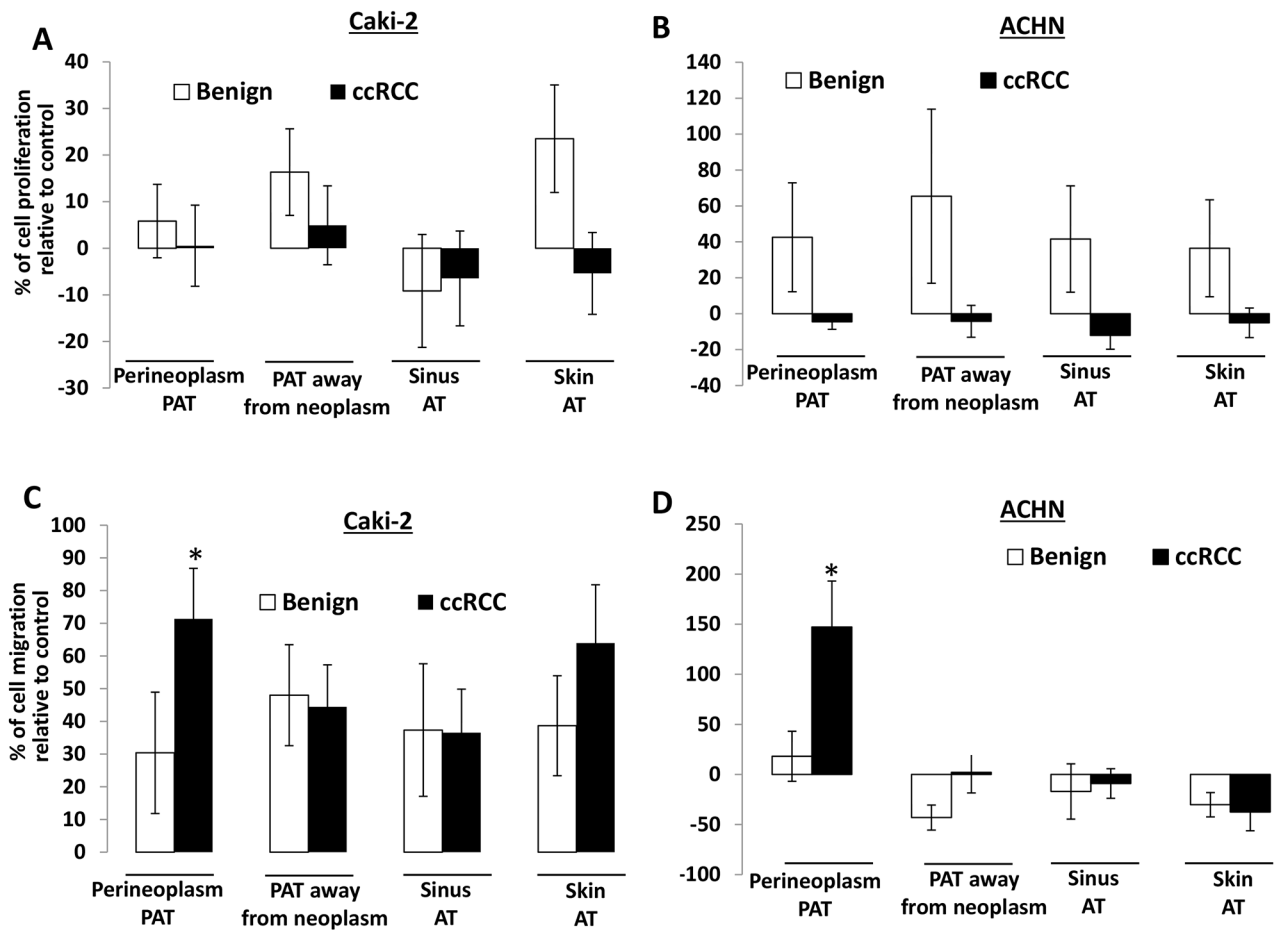
The effect of different AT CMs on proliferation and migration of Caki-2 and ACHN cells **A.** 2×10^4^ Caki-2 or ACHN cells were plated in 24-well culture plates. After 24 hours, the medium was changed to serum-free medium (negative control) or CMs from different AT cultures, including perineoplasm PAT, PAT away from neoplasm, renal sinus and skin ATs from patients with ccRCC or benign renal diseases. After 72 hours of incubation, cell densities were measured by MTT assay. Columns, mean percentages of cell proliferation under treatment of AT CM relative to negative control; bars, SEM. Experiments were replicated thrice. **B.** cells were applied to the upper surface of a membrane. After incubation for 48 hours, the upper surface of the membrane was scrubbed free of cells; the membrane was fixed, H&E stained, and photographed. Representative pictures were taken from the lower surface of three independent membranes at ×100 magnification. Number of migrated cells was counted from 10 random fields. Columns, mean percentages of migratory cells under treatment of different AT CM relative to negative control; bars, SEM. Experiments were replicated thrice. “*” denotes P < 0.05.

Figure 1A and 1B show that CMs of different AT cultures from ccRCC have no significant effect on the proliferation of Caki-2 and ACHN cells, whereas most CMs of different AT cultures from benign renal diseases increase the proliferation of Caki-2 and ACHN cells, except for a decrease in the proliferation of Caki-2 cells by CMs of sinus AT from benign renal diseases.

In contrast, CMs of different AT cultures all increase the migration of Caki-2 cells by 30 to 70% compared to the negative control (i.e. serum-free medium) (Figure 1C). For ACHN cells, only perineoplasm PAT CM shows ~150% increase in cell migration relative to the negative control, whereas CMs from other AT cultures (i.e. PAT away from neoplasms, sinus and skin ATs) either have no or decreased effect on cell migration (Figure 1D). Importantly, perineoplasm PAT CMs from ccRCC exhibit ~8.2 and ~2.4 fold higher capacity of promoting the migration of Caki-2 and ACHN cells, respectively, than those from benign renal diseases (*P*s<0.05) (Figure 1C and 1D). These results suggest that perineoplasm PAT may play a role in ccRCC progression.

### The effect of perineoplasm PATs from different tumor stages of ccRCC on the proliferation and migration of Caki-2 and ACHN cells compared to those from benign renal diseases

**Figure 2:**
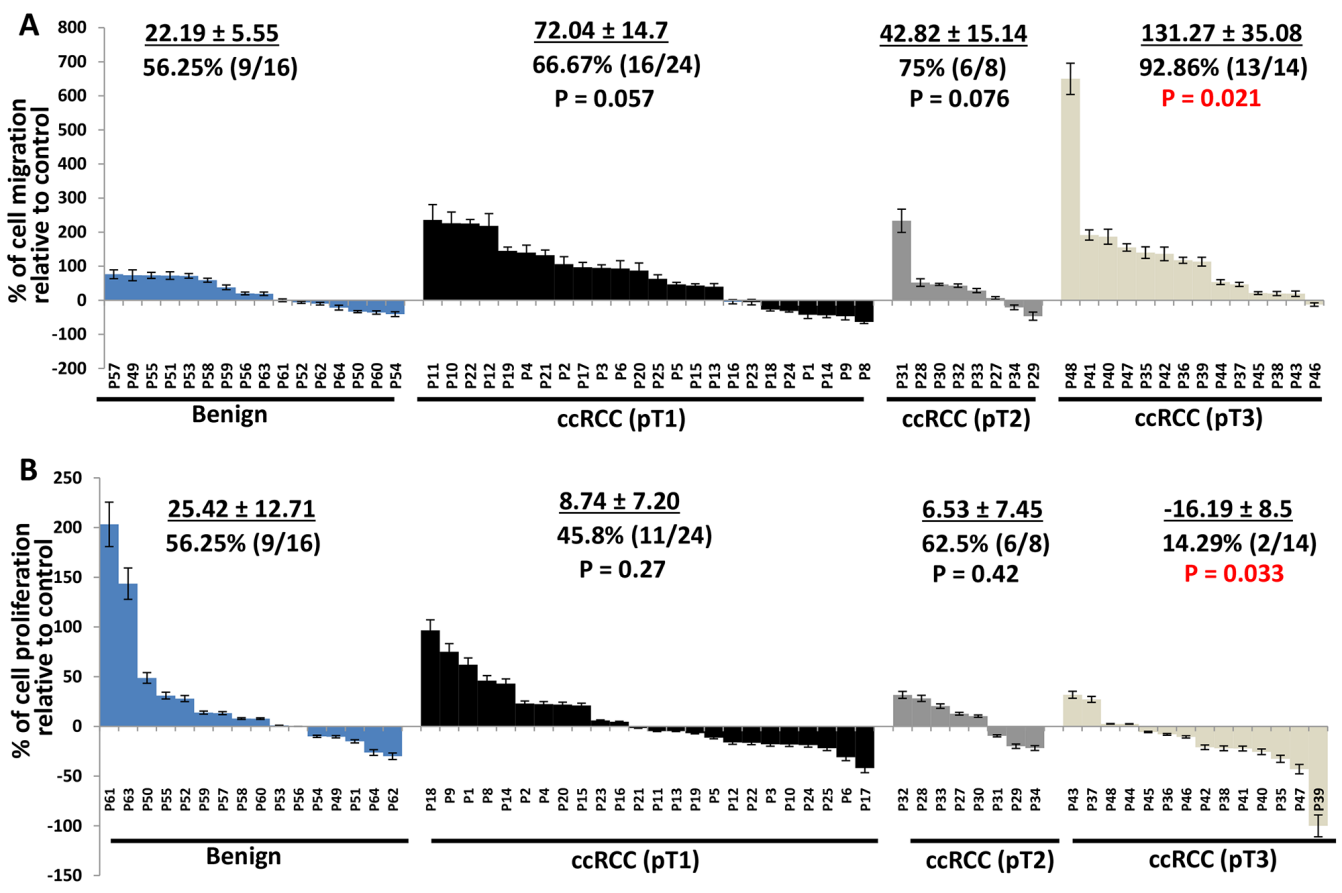
Waterfall plotting of percentage increase or decrease of perineoplasm PAT CMs that induced cell migration and proliferation **A.** Caki-2 cells were applied to the upper surface of a membrane. After incubation for 48 hours, the upper surface of the membrane was scrubbed free of cells; the membrane was fixed, H&E stained, and photographs were taken from the lower surface of three independent membranes at ×100 magnification. Migrated cells were quantified by counting 10 fields under the microscope. Columns, mean percentages of migratory cells under treatment of perineoplasm PAT CM relative to negative control; bars, SEM. P1,..,62 represent patient #1,…, 62. **B.** 2×10^4^ cells were plated in 24-well culture plates. After 24 hours, the medium was changed to serum-free medium (negative control) or perineoplasm PAT CM from patients with benign renal diseases, pT1, pT 2, and pT 3 ccRCC. After 72 hours of incubation, cell densities were measured by MTT assay. Columns, mean percentages of cell proliferation under treatment of PAT CM relative to negative control; bars, SEM. Experiments were replicated in triplicate.

We next examined whether the effect of perineoplasm PATs on cell proliferation and migration are associated with different tumor stages of ccRCC. Figure 2A shows the percentages of PAT CMs that promoted cell migration and how much percent increase of migrated cells relative to control in each patient. The percentages of CMs promoting migration progressively increased from benign to pT1, pT2, through pT3 ccRCC from 56.25% to 66.67%, 75%, and 92.82% respectively. The means of percent increase in migration relative to negative control in individual patients also increased from benign to pT1, pT2, through pT3 ccRCC from 22.19 ± 5.55, 72.04 ± 14.7, 42.82 ± 15.14, and 131.27 ± 35.08. Perineoplasm PAT CMs from pT3 ccRCC significantly increase the migration of ccRCC compared to those from benign renal diseases (*P* = 0.021). In contrast, the percentage of perineoplasm PAT CMs from pT3 ccRCC with higher cell proliferation capacity decreased. The means of percent increase in proliferation relative to negative control by perineoplasm PAT CMs in individual patients from pT3 RCC also significantly reduced compared to those from patients with benign renal diseases (-16.19 ± 8.5 vs. 25.42 ± 12.71; *P* = 0.033) (Figure 2B).

**Figure 3:**
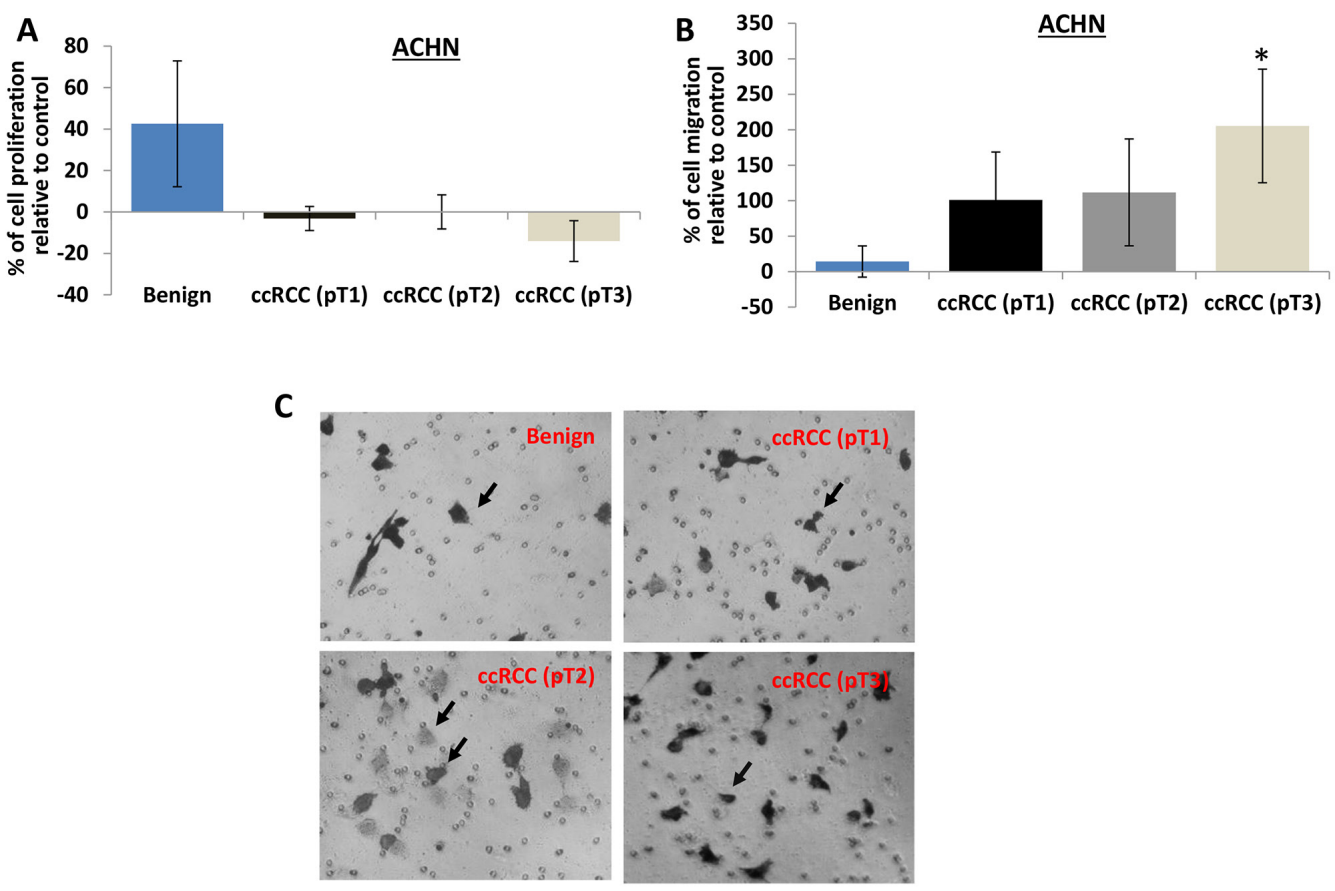
The effect of perineoplasm PAT CMs from patients with benign renal diseases or different tumor stages of ccRCC on proliferation and migration of ACHN cells **A.** the proliferation of ACHN cells was assayed as described above. Perineoplasm PAT CMs from patients with benign renal diseases increase the proliferation of ACHN cells (P<0.05), whereas perineoplasm PAT CMs from patients with pT3 ccRCC decrease the proliferation of ACHN cells (P<0.05). **B.** the migration of ACHN cells were examined as described above. Perineoplasm PAT CMs from patients with pT3 ccRCC significantly increase the migration of ACHN cells compared to those from patients with benign renal diseases or pT1 or pT2 ccRCC (Ps<0.05). **C.** representative pictures of migrated ACHN cells were taken from the lower surface of three independent membranes at ×100 magnification. Arrows indicate invaded cells.

Consistently, perineoplasm PAT CMs from pT3 ccRCC also increases migration of ACHN cells, but decreases their proliferation compared to those from patients with benign diseases (Figure 3, *Ps*<0.05).

These results suggest that secreted factors by perineoplasm PATs may be associated with more aggressive ccRCC by promoting cell migration.

### The effect of perineoplasm PAT CMs from different Fuhrman grades of RCC on the proliferation and migration of Caki-2 cells

Fuhrman grade is the most commonly used grading system for RCC prognosis; and higher Fuhrman grade suggests worse prognosis [18].

**Figure 4:**
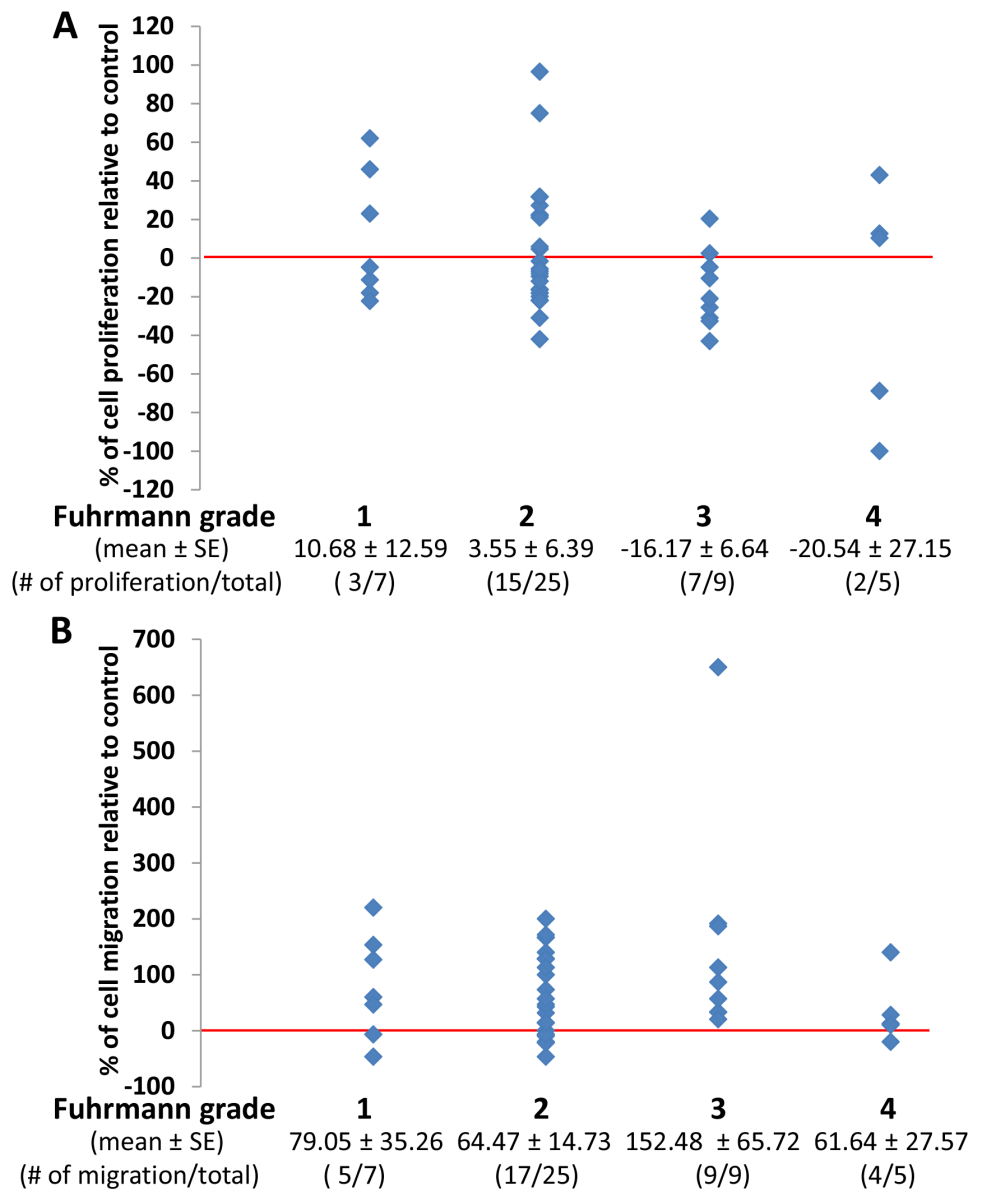
The comparison of the effect of perineoplasm PAT CMs from patients with different Fuhrman grades on proliferation and migration of Caki-2 cells **A.** there are no significant differences in mean percentages of cell proliferation under culture conditions of perineoplasm PAT CMs from patients with different Fuhrman grades. Each dot represents a percentage value of cell proliferation by perineoplasm PAT CMs from a patient with indicated Fuhrmann grade relative to negative control. **B.** perineoplasm PAT CMs from patients with higher Fuhrman grade are more likely to increase the migration of Caki-2 cells. Each dot represents a percentage value of cell migration by perineoplasm PAT CM from a patient with indicated Fuhrman grade relative to negative control.

Figure 4A shows that perineoplasm PAT CMs from 9 out of 14 (64.3%) patients with high Fuhrman grades (grade 3 and 4) of ccRCC compared to 18 out of 32 (56.3%) low-grade (grade 1 and 2) ccRCC patients inhibited the proliferation of Caki-2 cells. The means of percent increase in proliferation relative to negative control by perineoplasm PAT CMs in patients with high *versus* low Fuhrman grade ccRCC are -17.73 ± 9.94 vs. 5.11 ± 5.63.

In contrast, perineoplasm PAT CMs from 13 out of 14 (92.9%) patients with high Fuhrman grade 3 and 4 of ccRCC increased the migration of Caki-2 cells, whereas perineoplasm PAT CMs from 22 of 32 (68.8%) patients with low Fuhrman grade 1 and 2 of ccRCC promoted the migration of Caki-2 cells (Figure 4B). The means of percent increase in migration relative to negative control in patients with high *versus* low Fuhrman grade ccRCC are 110.26 ± 45.16 vs. 67.67 ± 13.60.

These results suggest that perineoplasm PAT CM from high Fuhrman grade ccRCC may be associated with decrease proliferation and increased migration of ccRCC cells.

### Perineoplasm PAT CMs from patients with larger tumor volume is associated with an inhibitory effect on the proliferation of Caki-2 cells, but not related to the migration of Caki-2 cells

Several studies have demonstrated that larger tumor size particularly in combination with perinephric fat invasion predicted worse cancer specific survival in RCC patients [19–21]. These results underline the importance of understanding the biological association of PAT and tumor size. We therefore evaluated the association between tumor volume and the effects of perineoplasm PAT CMs on the proliferation and migration of Caki-2 cells. In this study, tumor volume represents a more accurate measurement of tumors than tumor size.

**Figure 5:**
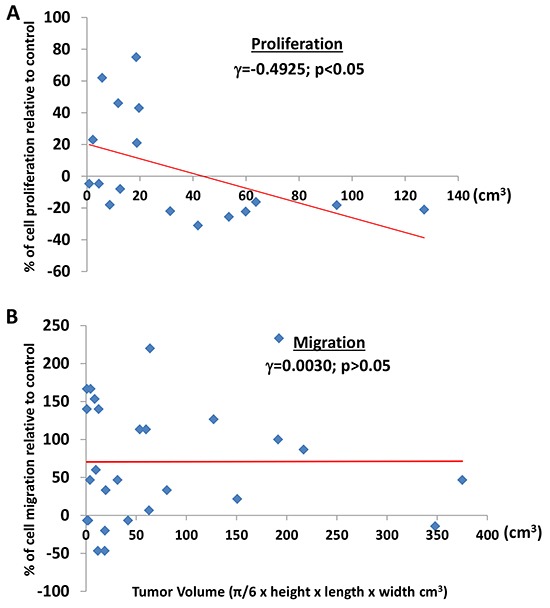
The correlation between tumor volume and the effect of perineoplasm PAT CMs on cell proliferation or migration **A.** tumor volume is associated with the inhibitory effect of perineoplasm PAT CMs on the proliferation of Caki-2 cells. Each dot represents a percentage value of cell proliferation by perineoplasm PAT CM from a patient with corresponding tumor volume. **B.** regardless of tumor volume, perineoplasm PAT CMs from the majority of patients increased the migration of Caki-2 cells. Each dot represents a percentage value of cell migration by perineoplasm PAT CM from a patient with corresponding tumor volume.

Figure 5A shows that perineoplasm PAT CM from 100% (7 out of 7) and about 40% (4 out of 10) of patients with tumor volume >20 cm^3^ and <= 20 cm^3^, respectively, inhibited the proliferation of Caki-2 cells. Perineoplasm PAT CM from patients with larger tumor volume is significantly associated with inhibitory activity toward the proliferation of Caki-2 cells (Figure 5A; γ = -0.4925, *P*<0.05).

Regardless of tumor volume, 74% (20 out of 27) perineoplasm PAT CMs increased migration of Caki-2 cells. Among them, perineoplasm PAT CM from 61.5% (8 out of 13) and 86% (12 out of 14) of patients with tumor volume ≤ 20 cm^3^ and >20 cm^3^, respectively, increased the migration of Caki-2 cells (Figure 5B). Perineoplasm PAT CM from the remaining two patients with larger tumors (>20 cm^3^) only minimally inhibited Caki-2 migration (approximately 6% and 14%). We did not note a relationship between the migratory capacity of perineoplasm PAT CM and tumor volume (Figure 5B; γ = 0.0030, *P*>0.05).

These results demonstrated that secreted factors from perineoplasm PATs of patients with large tumor volume may inhibit tumor growth but promote metastasis of ccRCC via increasing the mobility of ccRCC cells.

### The WNT/β-catenin activity of perineoplasm PAT CM from patients with different stages of ccRCC

**Figure 6:**
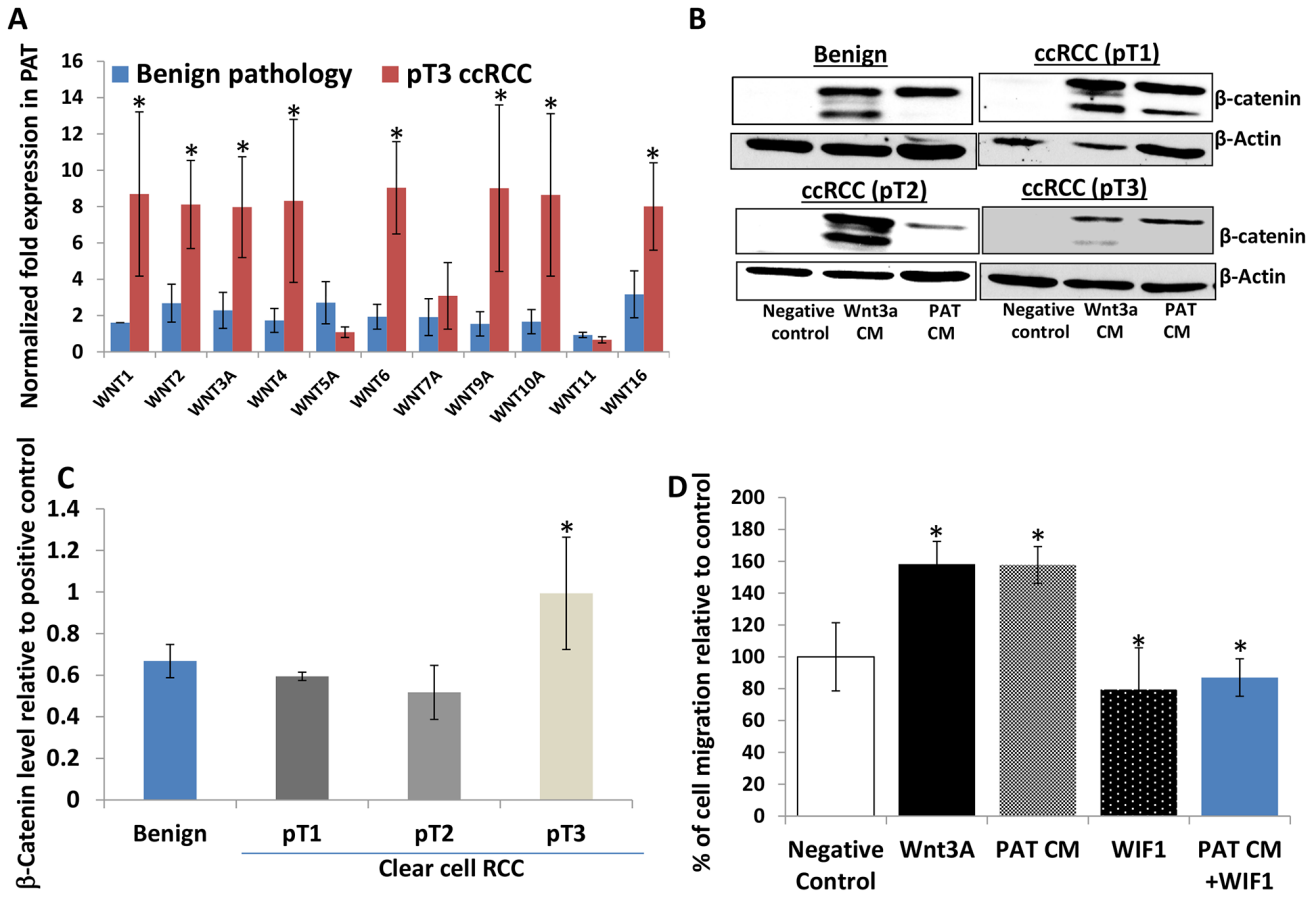
Perineoplasm PATs from patients with pT3 ccRCC overexpress multiple WNTs compared to those with benign renal neoplasms and their CMs induce accumulation of cytoplasmic β-catenin in L cells **A.** pT3 ccRCC PATs (n=6) express higher mRNA levels of WNTs compared to PATs from patients with benign renal neoplasms (n=6). **B.** Western blotting analysis of cytoplasmic β-catenin levels induced by negative control (serum-free medium), WNT3A CM, and PAT CMs from benign renal neoplasms or different tumor stages of ccRCC was shown by representative blots. β-Actin serves as a loading control. **C.** relative cytoplasmic β-catenin levels induced by PAT CMs from patients with benign renal neoplasms and ccRCC. Semi-quantitative measurement of cytoplasmic β-catenin levels by densitometry using NIH free software imaging J system and adjusted by the load control (β-actin). Relative β–catenin levels were calculated by dividing β–catenin levels stimulated by each PAT CM by those induced WNT 3A CM (positive control) on the same blot. Mean levels of relative β–catenin levels are shown; bars, SEM. pT3 ccRCC PAT CMs (n=12) exhibited higher WNT activity (measured by β–catenin levels) compared to benign (n=12) and pT1 (n=12) and T2 (n=8) ccRCC. **D.** the effect of the recombinant WIF1 protein on WNT 3A and PAT CMs induced migration of Caki-2 cells. Cells were applied to the upper surface of a membrane and PAT CMs with or without 0.29 μmol/L WIF1 recombinant protein or serum free medium (negative control) in the lower chamber as a source of chemoattractant. Percentages of migrated cells were calculated as described in Figure 1B legend and in Materials and Methods; bars, SEM. Experiments were replicated thrice.

Kruck and colleagues [22] have examined the expression of WNT1/β-catenin expression in 278 ccRCC patients, showing that cytoplasmic β-catenin was significantly associated with unfavorable clinic pathology and worse overall and worse cancer specific survival. Studies from other groups indicated that autocrine WNT ligand secretion and loss of secreted WNT antagonists by epigenetic silencing could contribute to the enhanced WNT activity in ccRCC tumors [17, 23, 24]. However, to date we have not identified a report regarding a potential role of paracrine WNT activity in PAT. As such, total RNAs were extracted from six matched pairs (by age and sex) of perineoplasm PATs from pT3 ccRCC versus benign neoplasms. The mRNA levels of 11 WNT ligands were investigated by quantitative PCR method. Figure 6A reveals that mRNA levels of 8 out of 11 WNTs (including WNT1, 2, 3A, 4, 6, 9A, 10A and 16) are about 2.3 to 9.5 fold higher in perineoplasm PATs from pT3 ccRCC than those from benign neoplasms (*Ps* < 0.05).

We also have examined the WNT/β-catenin activity of perineoplasm PAT CM from patients with different tumor stages of ccRCC *versus* benign neoplasms. β-Catenin is a dual function protein and it can regulate both cell-cell adhesion and WNT mediated gene activation [25, 26]. Membranous β-catenin acts primarily as a cell adhesion molecule [25, 26]. Therefore, L cells that lack membranous β-catenin are used to examine WNT mediated cytoplasmic β-catenin accumulation as a surrogate maker for WNT activity [26]. L cells were treated with perineoplasm PAT CMs and we performed Western blotting to analyze cytoplasmic β-catenin accumulation stimulated by negative control (serum free medium), WNT 3A CM (a positive control) and perineoplasm PAT CM, using the method as we have previously described [26]. The relative WNT/β-catenin activity of perineoplasm PAT CM was calculated by dividing levels of perineoplasm PAT CM induced cytoplasmic β-catenin by those induced by WNT 3A.

Figure 6B shows that L cells under serum free culture conditions express little or no cytoplasmic β-catenin, and that both WNT 3A and perineoplasm PAT CM regardless of patients' pathological status strongly induced accumulation of cytoplasmic β-catenin. We then performed semi-quantification of Western blotting band densities to calculate relative levels of cytoplasmic β-catenin to positive control (WNT 3A) treatment. Figure 6C demonstrates that the mean levels of cytoplasmic β-catenin induced by perineoplasm PAT CMs from patients with pT3 ccRCC are higher than those from patients with benign renal diseases or pT1 and pT2 ccRCC (*P*s<0.05).

The enhanced WNT/β-catenin activity in perineoplasm PAT CM from patients with pT3 ccRCC correlated with increased capacity of these CM to promote Caki-2 migration as we have shown in Figure 2A and Figure 6D.

Addition of 0.29 μmol/L Wnt inhibitory factor 1 WIF1 recombinant protein into pT3 ccRCC perineoplasm PAT CM completely blocked the PAT CM induced migration of Caki-2 cells (Figure 6D), which confirmed a role of WNT signaling in the perineoplasm PAT CM mediated migration.

Further studies are in progress to identify what secreted factors, including WNT ligands, antagonists and target genes, are present in perineoplasm PAT CM that could be responsible for promoting the migration of ccRCC cells by an unbiased proteomic approach. The identification and study of these secreted factors in perineoplasm PAT may help to develop future targets for ccRCC therapies and may lead to the discovery of a novel surrogate marker for predicting RCC histology and outcomes. Our study may be still limited by the relatively small sample size of RCC patients, which currently precludes a more comprehensive clinical or subgroup evaluation of different histological types of RCCs.

In addition, the mechanism for increased migration capacity of perineoplasm PAT CM vs. CM from PAT away from neoplasms remains unknown. It is possible that there may be a gradient effect of secreted Wnt factors on cell migration as reported in other cancer stroma interactions [27, 28]. Therefore, further studies will be set up to examine the expression of secreted Wnt factors at the interface between PAT and tumor or “normal” tissues and the relationship of RCC stages or cell migration with tumor/fat distance and areas of contact.

In conclusion, to the best of our knowledge, this is the first report about the biological effect of PAT secreted factors or CMs on ccRCC cell proliferation, migration, and WNT activity. Perineoplasm PAT CMs have higher capacity of promoting the migration of RCC cell lines Caki-2 and ACHN compared to CMs from other AT cultures, including PAT away from neoplasms, sinus and skin ATs. Perineoplasm PAT CM from pT3 ccRCC patients significantly promoted the migration of Caki-2 and ACHN, but inhibited their growth compared to those from patients with benign renal diseases. Consistent with this result, perineoplasm PATs express multiple WNTs from pT3 ccRCC patients and their CMs have higher WNT activities as measured by cytoplasmic β-catenin accumulation. Perineoplasm PAT CMs from patients with larger tumors is associated with inhibitory effects on the growth of Caki-2 cells. However, regardless of tumor size, stage and histological types, perineoplasm PAT CMs in general promoted migration of Caki-2 cells. Taken together these results demonstrate that secreted factors from perineoplasm PATs may play a role in facilitating metastasis or perirenal fat invasion of ccRCC by mobilizing ccRCC cells away from primary tumor sites. Further studies are in progress to identify potential secreted factors that are responsible for the effect of perineoplasm PAT CMs on ccRCC cell migration.

## MATERIALS AND METHODS

### Study population and surgical specimen collection

After receiving University of California-Irvine institutional review board approval, patients undergoing renal surgery (radical, simple, or partial nephrectomy) were consented to participate in the study. We prospectively collected clinical and pathological characteristics including age, sex, race, BMI, Fuhrman grade, pathological tumor size, and clinical stage. We calculated BMI as patient weight in kilograms divided by their height in meters squared. We processed surgical specimens by standard pathologic techniques and each specimen was reviewed by genitourinary pathologists. Tumors were staged according to the 2010 American Joint Committee on Cancer (AJCC) Tumor Nodes Metastasis (TNM) classification [29]. Patients undergoing renal surgery with urothelial cancers of the renal pelvis (n = 3) were excluded from this study. As were patients with chromophobe (n=7) or papillary tumors (n=1). As part of the standard procedure for renal surgery, perineoplasm PAT (fat tissue around ccRCC or around benign neoplasms), PAT (more than 3 cm away from the outer margin of the neoplasm) away from neoplasm, renal sinus and subcutaneous ATs was removed and collected.

### Collection of conditioned medium from AT cultures

Adipose tissue samples (typically ~1 g) were processed under a sterile laminar flow hood, where adipose tissues were minced into 20–80 mg pieces using scissors, and rinsed extensively in phosphate-buffered saline as previously described [30]. Red blood cells and other debris were removed by centrifuging for 1 minute at 277 × gravity. The clean adipose tissues were then cultured in M199 (Invitrogen) culture medium supplemented with 50 μg/ml gentamicin per gram of tissue for 24 hours. CM was collected and aliquoted into 1 ml fractions and was immediately analyzed or stored at -80°C. CM was used for testing the effect of secreted factors from adipose tissues on cell growth and cell migration and for assaying WNT activity.

### MTT assay for evaluating cell proliferation [31]

Caki-2 and ACHN were purchased from American Type Culture Collection (ATCC) (Manassas, VA) and used within 20 passages after receipt. The cell lines were tested and authenticated by ATCC. Caki-2 and ACHN cells were maintained in McCoy's 5A and Eagle's Minimum Essential Medium (EMEM) growth medium, respectively, with 10% fetal bovine serum (FBS) and 1% pen/strep added. Cells at a density of 2 × 10^4^ per well in 24-well plates were plated in culture medium containing 10% FBS. After 24 hours, the medium was refreshed with fresh serum-free medium or CM from AT culture. After 72 hours, MTT was added to each well at a final concentration of 1 mg/mL and incubated at 37°C for 2 hours. The media/MTT solution was then removed without disturbing the attached cells, and accumulated formazan in cells dissolved in acidified (4% 1N HCl) isopropanol. The absorbance of each sample was determined at 570 nm. Each sample was repeated three times. The percentage of cell proliferation relative to negative control (cultured in serum free condition) was calculated.

### Cell migration and recombinant WIF1 protein treatment

Cell migration assay was performed using 24-well Boyden chamber system (BD Biosciences; Franklin Lakes, New Jersey) as described previously [26]. Caki-2 and ACHN cells were cultured in serum-free medium for 24 hours. After trypsinization, 2.5 x 10^4^ Caki-2 or ACHN cells per well were placed in the upper chamber. WIF1 recombinant protein was generated and tested for WNT inhibitory activity as previously described [26]. CM was placed with or without WIF1 recombinant protein or 1% FBS (positive control) or serum free medium (negative control) in the lower chamber as a chemoattractant. Cells were allowed to migrate through a porous, uncoated membrane (BD Biosciences) for 24 hours at 37 ºC. We removed non-migratory cells in the upper chamber with a cotton-tip applicator. Migrated cells on the lower surface were fixed with methanol and stained with hematoxylin. The mean number of migrating cells was determined after counting cells on 10 high-power fields (x100) on each membrane. The percentage of migration induced by CM relative to negative control was calculated. Each experiment was repeated three times.

### Quantitative RT-PCR

Total RNAs were extracted from six matched pairs (by age and sex) of perineoplasm PATs from pT3 ccRCC versus benign neoplasms and the mRNA levels of 11 WNT ligands were examined by real-time quantitative PCR amplification using the CFX96 Touch Real-Time PCR Detection System (Bio-Rad) as described previously [25, 26]. The sequences of primers for WNT1, 2, 3A, 4, 5A, 6, 7A, 9A, 10A, 11 and 16 are available upon request. Data were analyzed by using the comparative Ct method, where Ct is the cycle number at which fluorescence first exceeds the threshold. The Ct values from each sample were obtained by subtracting the values for β-actin Ct from the target gene Ct value. The variation of β-actin Ct values is <0.5 among different samples. A one cycle difference of Ct value represents a 2-fold difference in the level of mRNA. Specificity of resulting PCR products was confirmed by melting curves and agarose gel.

### WNT/β-catenin activity

WNT/β-catenin activity in PAT CM was examined as induced levels of β -catenin. L-cells were cultured in PAT CM, WNT 3A CM (positive control) or serum-free medium (negative control) for 24 hours and then the cells were lysed in RIPA lysis buffer containing protease inhibitor cocktails [25]. Clarified protein lysates (30μg) were electrophoretically resolved on 8% denaturing SDS-polyacrylamide gel, transferred to nitrocellulose membranes, and probed with antibodies against β-catenin (BD Biosciensces; Franklin Lakes, New Jersey). After washing twice with TBST for 5 minutes each, proteins were detected using secondary antibodies (Santa Cruz Biotech; Santa Cruz, CA). The blots were visualized using Thermo Scientific Super-signal West Dura kit (Thermo Scientific). For a loading control, the membrane was placed in stripping buffer for 5 minutes to remove the primary and secondary antibodies. After washing with TBST for 5 minutes and blocking with 5% milk for 1 hour, the membrane was reprobed with β-actin antibody and secondary antibody and then visualized as previously described. β–Catenin levels were semi-quantified by densitometry using the free NIH software image J and adjusted by the loading control (β-actin). Relative β–catenin levels were calculated by dividing β–catenin levels stimulated by each PAT CM by those induced WNT 3A CM (positive control) on the same blot.

### Statistical analysis

Microsoft Excel software was used to compute the mean and standard deviations and standard errors of all quantitative data. We compared cell viability and migration between CM from patients with benign renal diseases and from those with ccRCC using either analysis of variance (ANOVA), Fisher exact or Student's t-test. All statistical measures were two-sided, and P-values <0.05 were considered to be statistically significant.
